# First-Pass Reperfusion by Mechanical Thrombectomy in Acute M1 Occlusion: The Size of Retriever Matters

**DOI:** 10.3389/fneur.2021.679402

**Published:** 2021-06-22

**Authors:** Carmen Serna Candel, Marta Aguilar Pérez, Hansjörg Bäzner, Hans Henkes, Victoria Hellstern

**Affiliations:** ^1^Neuroradiologische Klinik, Klinikum Stuttgart, Stuttgart, Germany; ^2^Neurologische Klinik, Klinikum Stuttgart, Stuttgart, Germany; ^3^Medical Faculty, University Duisburg-Essen, Essen, Germany

**Keywords:** first-pass, stroke, thrombectomy, reperfusion, stent retriever, large vessel occlusion, pRESET

## Abstract

**Introduction:** Single-pass complete reperfusion using stent retrievers has been shown to improve functional outcome in patients with large vessel occlusion strokes. The aim of this study was to investigate the optimal size of stent retrievers to achieve one-pass complete reperfusion by mechanical thrombectomy.

**Methods:** The study evaluated the results of aspiration-assisted mechanical thrombectomy of acute isolated occlusion of the middle cerebral artery in the M1 segment with a novel 5 × 40-mm stent retriever compared to the usual 4 × 20-mm device. Reperfusion status was quantified using the Thrombolysis In Cerebral Infarction (TICI) scale. We hypothesized that thrombectomy of M1 occlusions with 5 × 40-mm stent retriever yields higher rates of complete first-pass reperfusion (FP) (TICI ≥2c after one pass) and successful or modified FP (mFP) (TICI ≥2b after one pass) than thrombectomy with 4 × 20. We included isolated M1 occlusions treated with pRESET 5 × 40 (phenox) as first-choice device for thrombectomy and compared with M1 occlusions treated with pRESET 4 × 20. We excluded patients with additional occlusions or tandem stenosis or who received an intracranial stent or angioplasty as a part of the endovascular treatment.

**Results:** One hundred thirteen patients were included in the 4 × 20 group and 57 patients in the 5 × 40 group. The 5 × 40 group achieved higher FP compared to 4 × 20 group [61.4% (35 of 57 patients) vs. 40.7% (46 of 113), respectively; adjusted odds ratio (OR) and 95% confidence interval (95% CI) = 2.20 (1.08–4.48), *p* = 0.030] and a higher mFP [68.4%, 39 of 57 patients vs. 48.7%, 55 of 113; adjusted OR (95% CI) = 2.11 (1.04–4.28), *p* = 0.037]. Frequency of successful reperfusion (TICI ≥2b) was similar in both groups (100 vs. 97.3%), but frequency of complete reperfusion (TICI ≥2c) was higher in the 5 × 40 group [82.5 vs. 61.9%, adjusted OR (95% CI) = 2.47 (1.01–6.04), *p* = 0.047]. Number of passes to achieve reperfusion was lower in the 5 × 40 group than in the 4 × 20 group [1.6 ± 1.1 vs. 2 ± 1.4, *p* = 0.033; adjusted incidence rate ratio (95% CI) = 0.84 (0.69–1.03), *p* = 0.096]. Modified Rankin scale at 90 days was similar in 5 × 40 and 4 × 20 groups.

**Conclusions:** The size of stent retriever matters in acute M1 occlusions treated with aspiration-assisted mechanical thrombectomy. A longer stent retriever with a larger nominal diameter achieves a higher complete and successful FP and higher successful reperfusion compared to a shorter stent retriever.

## Introduction

Mechanical thrombectomy has become standard of care for patients with acute ischemic stroke and large vessel occlusion (LVO). The goal of the thrombectomy is to achieve reperfusion as early as possible to maximize the probability of good functional clinical outcome (1). Although a successful reperfusion, measured after endovascular treatment as a Thrombolysis In Cerebral Infarction (TICI) ≥2b score in the angiography, was the initial goal, a complete reperfusion TICI 3 is associated with greater neurological improvement during hospitalization, better functional outcome at 90 days, and reduced infarct growth (2, 3); moreover, increasing degrees of reperfusion associate better clinical outcome (4). Therefore, achieving TICI 2c or 3 should be the new aim of mechanical thrombectomy for anterior circulation (5).

The first-pass effect (FPE), restoring a complete or near-complete reperfusion (TICI 2c-3) in a single pass, is an independent predictor of good clinical outcome (6–8). FPE is also associated with lower healthcare resources use and lower estimated costs (8), and therefore, it should be pursued as the new goal of the endovascular treatment of LVO with thrombectomy devices. Thrombectomy with stent retriever under aspiration has shown higher rates of first-pass reperfusion (FP) (9) or similar (10) compared to direct aspiration.

There are no recommendations regarding size selection of stent retriever for thrombectomy, and this remains at the discretion of the neurointerventionalist. Larger devices have shown *in vitro* a higher frequency of complete clot removal (11, 12). Use of longer stent retrievers (30–40 mm) was found to be an independent predictor of FP in internal carotid artery (ICA) and middle cerebral artery (MCA) occlusions in comparison to shorter stent retrievers (20 mm) by equal diameter, enhancing the idea that longer retrievers offer a larger surface area of interaction with the thrombus reducing the possibility of leaving a clot behind (13). A longer stent retriever (4 × 40) showed the highest frequency of FP compared with larger diameter (6 × 30) and shorter stents (4 × 20) in ICA, MCA-M1, and MCA-M2 occlusions (14). *In vitro*, it was reported that longer stent retrievers (6 × 40 vs. 6 × 30) achieve higher FP in fibrin-rich clots (15). On the contrary, one study comparing diameter (4 vs. 6 mm) found no difference in reperfusion rate of ICA, MCA, and anterior cerebral artery (ACA) occlusions (16), and others found higher rates of modified FP (mFP) (TICI ≥2b) in the MCA with short stent retrievers (20 mm vs. others) (17).

Our aim is to describe results of thrombectomy of acute isolated M1-MCA occlusions with a new longer size of stent retriever (5 × 40) compared to the usual size used in our center (4 × 20) (18). We hypothesized that thrombectomy of M1 occlusions with a 5 × 40 stent retriever is associated with higher rates of angiographic FP than thrombectomy with a 4 × 20 stent retriever.

## Materials and Methods

### Patient Population

Using a prospective stroke registry, where we collect all the patients referred to our interventional neuroradiology department in order to receive endovascular treatment, we identified the patients with isolated occlusion of MCA in M1 segment treated with pRESET 5 × 40 (phenox, Bochum, Germany), with 5-mm diameter and 40-mm length as first-choice device. The new size of stent retriever is also recommended, as 4 × 20, for 2-mm vessel diameter and was available in our center from December 2019. Until then, we had used pRESET 4 × 20, with 4-mm diameter and 20-mm length as standard stent retriever for thrombectomy in M1 occlusions. Since November 2019, both sizes had been used in our center for thrombectomy in M1 occlusions at the discretion of the neurointerventionalists. We compared patients with acute M1 occlusions treated with 5 × 40 as first-choice device with those patients with M1 occlusion treated with 4 × 20 as first-choice device the previous year (December 2018 to November 2019). For the period where both sizes were available, there was a tendency to use the size 5 × 40. Therefore, we did not find comparable patients treated with the size 4 × 20 during this period, suggesting a selection bias could occur. We excluded patients with additional occlusions or tandem stenosis or who received an intracranial stent or angioplasty as a part of the endovascular treatment.

The new size of stent retriever pRESET 5 × 40 was used between November 2019 and October 2020 in 89 patients with MCA occlusions: 9 were M2-MCA occlusions, and 80 were M1-MCA occlusions. From eligible M1-MCA occlusions: 57 were included in the present study (representing group 5 × 40), and 23 were excluded: 11 because of additional occlusions (9 tandem M1-ICA, 1 tandem A2-M1-ICA, 1 A2-M1) and 12 because an intracranial stent and/or angioplasty was needed (10 patients: proved intracranial stenosis, 2 patients: dissection after thrombectomy or intracranial stenosis). For the comparison group of M1-MCA occlusions treated with pRESET 4 × 20, we selected all the consecutive patients between December 2018 and November 2019. From 126 M1 occlusions treated over the mentioned period with 4 × 20: 113 were included (representing group 4 × 20), and 13 were excluded: 3 because of additional occlusion, 5 because pRESET LT was used, and 5 because intracranial stenting and/or angioplasty was performed.

Our hospital is a certified comprehensive stroke center, providing endovascular service for 13 regional stroke units. The local ethics committee approved the data collection and analysis. We treat patients initially presented to our hospital or patients referred from other hospitals [with or without previous intravenous (IV) recombinant tissue plasminogen activator (rtPA)]. In our center, all stroke patients with LVO are eligible for mechanical thrombectomy. If there are no contraindications, IV rtPA is administered in eligible patients, according to clinical guidelines (1). We perform computed tomography (CT), CT angiography (CTA), and CT perfusion. Presence or absence of mismatch in CT perfusion is informative and does not preclude the treatment. In the case of unknown onset time, magnetic resonance (MR) with MR angiography (MRA) is the preferred imaging modality. Patients referred from other centers receive CT-CTA or MR-MRA. Patients with acute LVO are considered for endovascular treatment under real life conditions, without exclusion regarding age, baseline National Institute of Health Stroke Scale, time of stroke onset, comorbidities, baseline functional status prestroke, or Alberta Stroke Program Early CT Score (ASPECTS; all included patients had ASPECTS ≥4) as long as the joint assessment of neurology and neuroradiology proposed a realistic chance for improvement.

Endovascular treatment is performed with a standardized technique: 8F sheath, 8F guiding catheter, thrombectomy with stent retriever pRESET (phenox) in a 0.021-inch inner-diameter microcatheter [usually Trevo 18 (Stryker) or Velocity (Penumbra)] under proximal aspiration with a distal access catheter (DAC), such as Sofia (MicroVention) or ACE68 (Penumbra) catheters. A VacLok AT Vacuum Pressure Syringe (Merit Medical) aspirates through DAC during thrombectomy maneuver, when we advance DAC over the stent retriever at the same time that thrombectomy is undergone. After removal of the stent retriever, the DAC is left in M1 or M2 segments under aspiration and after a while removed. Sometimes the DAC and stent retriever are removed together under aspiration through the guiding catheter. In the 5 × 40 group, we used as first DAC Sofia Plus 6F (*n* = 30; 52.6%), Sofia 5F (*n* = 24; 42.1%), and pHLO (phenox) (*n* = 3; 5.3%), and in the 4 × 20 group, Sofia 5F (*n* = 83; 73.5%), Sofia Plus 6F (*n* = 27; 23.9%), and ACE68 (*n* = 3; 2.7%). First DAC used differed between groups (*p* < 0.001). No balloon guiding catheter (BGC) was used. When more passes were necessary, another DAC may have been used at the discretion of the neurointerventionalist. The size of the first stent retriever used was pRESET 5 × 40 mm in the 5 × 40 group and pRESET 4 × 20 mm in the 4 × 20 group. When more than one pass was needed, it could be done with the same stent retriever or with another one according to the preference of the operator, always according to anatomy and technical need. Other stent retrievers used were registered. Intra-arterial (i.a.) rtPA after thrombectomy was allowed: 2 patients (3.6%) with right M1 occlusions received i.a. rtPA after thrombectomy, both in the 5 × 40 group. All procedures were done under general anesthesia. Procedural experience and skills were considered similar between the neurointerventionalists.

Data on demographics, prestroke functional status [quantified by the modified Rankin scale (mRS)], and vascular risk profile were collected. The National Institute of Health Stroke Scale (NIHSS) score before angiography was considered as baseline neurological status. Last time seen well was considered stroke onset if time was unknown or in wakeup stroke. Stroke cause was defined, according to TOAST (Trial of ORG 101172 in Acute Stroke Treatment) classification (19).

### Imaging, Clinical, and Angiographic Assessment

Presence of early ischemic changes on admission CT or MRI [diffusion-weighted imaging (DWI)] and in control CT was assessed using the ASPECT score (20). Vessel occlusion was confirmed in primary imaging (CTA or MRA) and in the diagnostic run of the angiography. For patients transferred from other centers, primary imaging and time of imaging were considered from imaging of the referring center. We repeat the imaging after transfer to our center if hemorrhage is suspected by a clinical deterioration of ≥4 points in NIHSS. Occlusion of the MCA was differentiated into proximal, when thrombus was seen in proximal or middle segment of M1 segment of MCA and distal, when thrombus was seen in the distal third of the MCA with distal or no lenticulostriate arteries or in MCA bifurcation (21). Origin of the anterior temporal branch from M1 segment was still considered M1 occlusion. Collateral leptomeningeal status was assessed visually in CTA as previously described (22) and graded as follows: grade 1 = absent collaterals, grade 2 = less than the contralateral unaffected side, grade 3 = equal to the contralateral unaffected side, grade 4 = more than the contralateral unaffected side, and grade 5 = exuberant. The scale was dichotomized in “poor collaterals,” with less collaterals than contralateral unaffected side (grades 1–2), and “good collaterals,” with equal or greater collaterals to contralateral unaffected side (grades 3–4, as no case was graded as 5). In the angiography first run of the affected side, collaterals were assessed according to the American Society of Interventional and Therapeutic Neuroradiology/Society of Interventional Radiology (ASITN/SIR) scale (23) graded as grade 0: no collaterals visible at the ischemic site; grade 1 = slow collaterals to the periphery of the ischemic site with persistence of some of the defects; grade 2 = rapid collaterals to the periphery of the ischemic site with persistence of some of the defects and to only a portion of the ischemic territory; grade 3 = collaterals with slow but complete angiographic blood flow of the ischemic bed by the late venous phase; and grade 4 = complete and rapid collateral blood flow to the vascular bed in the entire ischemic territory by retrograde perfusion. The five grades were dichotomized as score 0–2 “poor collaterals” and score 3–4 “good collaterals.” If the first run was not performed until the end of venous phase, it was considered not valuable; this occurred in one case in the study.

Angiographic revascularization was assessed using the modified Thrombolysis in Cerebral Infarction (TICI) score in the final run of the angiography, measuring reperfusion in downstream territory of the specific arterial occlusion, as follows: grade 0 = no reperfusion; grade 1 = antegrade reperfusion past the initial occlusion, but limited distal branch filling with little or slow distal reperfusion; grade 2a = antegrade reperfusion of less than half of the occluded target artery previously ischemic downstream territory; grade 2b = antegrade reperfusion of more than half, but < 90% complete antegrade reperfusion; grade 2c = near-complete reperfusion (90–99%) except for slow flow in a few distal cortical vessels or presence of small distal cortical emboli; and grade 3 = complete antegrade reperfusion, with an absence of visible occlusion in all distal branches (4, 24, 25). TICI ≥2b was considered successful reperfusion, and TICI ≥2c was considered complete reperfusion. A reader reviewed all study cases under blinded conditions and compared the score with the score given the day of treatment. In the cases of discrepancy, a third blinded reader decided the score.

FP complete reperfusion was defined as achieving a complete reperfusion (TICI ≥2c) with a single thrombectomy device pass (6, 14). TICI 2c was included with TICI 3, as substantial evidence suggests that patients with LVO and a TICI 2c reperfusion after treatment follow the same clinical course as patients with a TICI 3 reperfusion (4, 5, 26). FPE was described in patients with a complete TICI 3 reperfusion after a single pass of thrombectomy, but patients with TICI 2c reperfusion were also included in the complete reperfusion category (6), with higher rates of good clinical outcome. mFP or successful FP was defined as achieving successful reperfusion (TICI ≥2b) after a single pass. The numbers of thrombectomy passes were recorded. Vasospasm after thrombectomy, dissection, or perforation was also recorded.

Furthermore, for each patient, we noted the following values: time from stroke onset to IV rtPA, time to groin puncture (stroke onset up to groin puncture), and time to recanalization (time to first assessment of final recanalization). Duration of treatment was the time from groin puncture to last run of angiography.

The clinical outcome was assessed as severity of disability at 90 days according to the mRS. An mRS ≤2 was considered a good clinical outcome (27). Further rates that were recorded were mortality at 90 days, subarachnoidal hemorrhage (SAH), and parenchymal hematoma (PH) [according to ECASS Classification (28)] in imaging 24–36 h after stroke or symptomatic intracranial hemorrhage (SICH) confirmed on neuroimaging (CT or MRI). A SICH was defined as any type of intracerebral hemorrhage on posttreatment imaging with an increase of at least 4 points on NIHSS (ECASS II) (28).

The primary outcome of our study was frequency of FP and mFP. Secondary outcomes were frequency of complete reperfusion (TICI ≥2c), number of passes of thrombectomy, and favorable clinical outcome at 3 months.

### Statistical Analysis

Metric variables were reported as mean with standard deviation (SD) or median with interquartile ranges; categorical variables were described by frequencies. Fisher exact test was used for testing the null hypothesis that two categorical variables were independent. Mann–Whitney *U*-test was used to compare whether two groups differed regarding the distribution of a metric variable.

For bivariate outcomes, logistic regression was used to determine which characteristics influence the likelihood of an event happening and Poisson regression to determine the variables with an influence on N passes. For multivariate analysis, variables that show a significant influence on the outcome in the bivariate analysis were chosen (full model). In addition, forward selection with a *p*-value threshold of 0.05 was used for further selection of variables in the multivariate analysis.

The analysis was performed with Stata/IC 16.1 for Unix, and the level of significance was set at a 0.05 level (two-sided).

## Results

**Figure 1:**
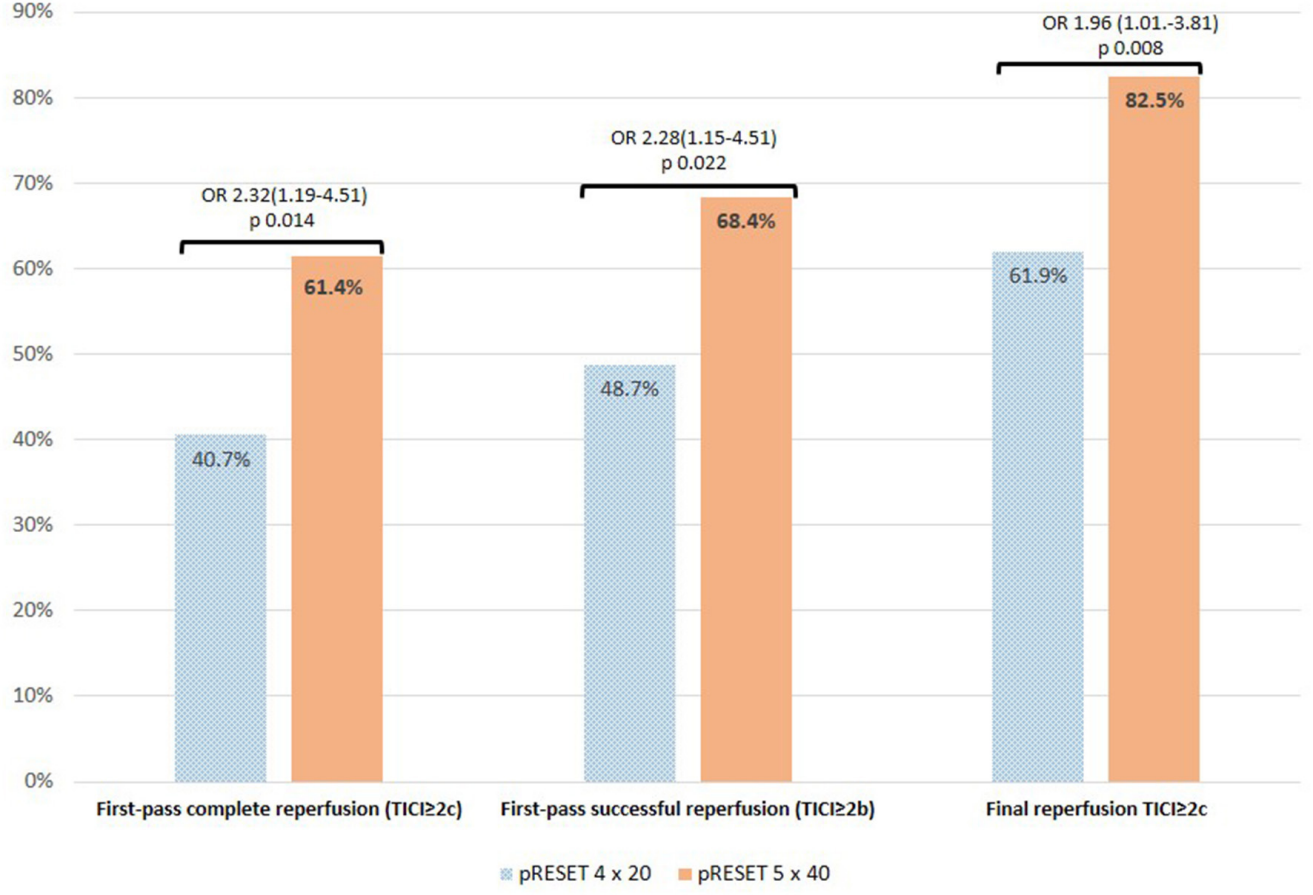
pRESET 5 × 40 vs. pRESET 4 × 20 in M1-MCA occlusions: primary and principal secondary outcomes. Abbreviations: M1-MCA, M1 segment of middle cerebral artery; TICI, modified thrombolysis in cerebral infarction score; first-pass complete reperfusion (TICI ≥2c) and first-pass successful reperfusion (TICI ≥2b) are abbreviated in text as FP and mFP, respectively. Statistics: Fisher exact test; odds ratio (OR) and 95% confidence interval (CI).

A total of 113 patients were included in the 4 × 20 group and 57 patients in the 5 × 40 group. Baseline characteristics between the 4 × 20 and 5 × 40 groups, including age, gender, stroke etiology, risk factors, mRS prestroke, NIHSS, ASPECTS, collateral status in CTA and in angiography, and IV rtPA treatment, among others, were comparable and are described in Table 1. As shown in Figure 1 and Table 2, the 5 × 40 group achieved significantly higher FP (61.4%, 35 of 57 patients) compared to the 4 × 20 group (40.7%, 46 of 113), unadjusted odds ratio (OR) [95% confidence interval (95% CI)] = 2.32 (1.19–4.51), *p* = 0.014, and adjusted OR = 2.20 (1.08–4.48), *p* = 0.030 (Table 3). Also the 5 × 40 group achieved a higher mFP (68.4%, 39 of 57 patients vs. 48.7%, 55 of 113), with unadjusted OR = 2.28 (1.15–4.53), *p* = 0.022, and adjusted OR = 2.11 (1.04–4.28), *p* = 0.037. Frequency of successful reperfusion (TICI ≥2b) was similar in both groups (4 × 20 vs. 5 × 40, 97.3% vs. 100%), but the frequency to achieve complete reperfusion (TICI ≥2c) was higher in the 5 × 40 group [4 × 20 vs. 5 × 40 (61.9% vs. 82.5%)] with unadjusted OR = 2.89 (1.30–6.43), *p* = 0.008, and adjusted OR = 2.47 (1.01–6.04), *p* = 0.047. Multivariate analysis is shown in Table 3.

**Table 1:** pRESET 5 × 40 vs. pRESET 4 × 20 in M1-MCA occlusions: baseline characteristics.

	**pRESET 4 × 20** **(*n* = 113)**	**pRESET 5 × 40** **(*n* = 57)**	*p*-value^*^
Age (years), mean ± SD	77.2 ± 11.6	77.6 ± 13	0.513
Gender male/female, n (%)	46 (40.7)/67 (59.3)	27 (47.4)/30 (52.6)	0.417
Cardioembolic etiology of stroke, n (%)	80 (70.8)	39 (68.4)	0.789
AF, n (%)	75 (66.4)	35 (61.4)	0.610
DM, n (%)	25 (22.1)	12 (21.1)	1
Hypercholesterolemia, n (%)	15 (13.3)	9 (15.8)	0.648
Smoker, n (%)	6 (5.3)	6 (10.5)	0.220
Hypertension, n (%)	74 (65.5)	42 (73.7)	0.301
Cardiovascular disease, n (%)	48 (42.5)	24 (42.1)	1
mRS prestroke: mRS ≤ 2/mRS >2, n (%)	89 (78.8)/24 (21.2)	44 (77.3)/13 (22.7)	0.377
Secondary transport, n (%)	82 (72.6)	45 (78.9)	0.456
Unknown onset, n (%)	42 (37.2)	19 (33.3)	0.735
NIHSS, median [IQR]	14 [11–18]	16 [11–19]	0.193
ASPECTS on baseline, median [IQR] Min–max	9 [8–10] 5–10	9 [8–10] 4–10	0.597
Collaterals CTA			0.103
Grade 1–2: poor collaterals, n (%)	34 (37.8)	25 (53.2)	
Grade 3–4: equal or greater collaterals, n (%)	56 (62.2)	22 (46.8)	
Collaterals angiography			0.241
Grade 0–2: poor collaterals, n (%)	45 (39.8)	17 (30.4)	
Grade 3–4: good collaterals, n (%)	68 (60.2)	39 (69.6)	
M1-MCA occlusion site			
Proximal/distal, n (%)	58 (51.3)/55 (48.7)	35 (61.4)/22 (38.6)	0.254
Left/right, n (%)	51 (45.1)/62 (54.9)	28 (49.1)/29 (50.9)	0.517
IV rtPA, n (%)	40 (35.4)	27 (47.4)	0.139
Time onset to IV rtPA, h, median [IQR]	1.8 [1.2–2.7]	1.7 [1.4–3.2]	0.706
Time imaging-groin puncture, h, median [IQR]	2.1 [1.8–2.7]	2.4 [2–2.8]	0.187
Time onset-groin puncture, h, median [IQR] Min–max	4.2 [3.1–7.4] 1.9–29.2	4.1[3–6.1] 2–13.4	0.513
Time to recanalization, h, median [IQR]	5 [3.8–7.8]	5.1 [3.8–7.2]	0.869
Duration of treatment, h, median [IQR]	0.6 [0.4–0.9]	0.6 [0.4–1]	0.427

* *Fisher exact test (qualitative variables); Mann–Whitney U-test (quantitative variables)*.

*AF, atrial fibrillation; ASPECTS, Alberta Stroke Program Early Computed Tomography Score on baseline imaging; CTA, computed tomography angiography; DM, diabetes mellitus; IQR, interquartile range; MCA, middle cerebral artery; min–max, minimum and maximum; mRS, modified Rankin scale; NIHSS, National Institute of Health Stroke Scale; IV rtPA, intravenous recombinant tissue plasminogen activator; SD, standard deviation; secondary transport, patients with LVO referred from other hospitals (with or without previous IV rtPA)*.

**Table 2:** pRESET 5 × 40 vs. pRESET 4 × 20 in M1 occlusions: safety parameters and clinical and angiographic outcomes.

	**pRESET 4 × 20 (*n* = 113)**	**pRESET 5 × 40 (*n* = 57)**	*p*-value^*^ OR (95% CI)
**Safety and imaging**			
ASPECTS post, median [IQR], min–max	7 [5–9] 0–10	7 [5–8] 1–10	0.342
PH, n (%)	5 (4.5)^§^	0	0.168
SICH, n (%)	0^§^	0	—
SAH, n (%)	18 (16.2)^§^	6 (10.5)	0.361
Vasospasm, n (%)	31 (27.4)	13 (22.8)	0.581
**Primary outcome**			
First-pass reperfusion (TICI ≥2c), n (%)	46 (40.7)	35 (61.4)	**0.014** 2.32 (1.19–4.51)
First-pass reperfusion (TICI ≥2b), n (%)	55 (48.7)	39 (68.4)	**0.022** 2.28 (1.15–4.53)
**Secondary outcomes**			
No. of passes, mean ± SD, min–max	2 ± 1.4, 1–9	1.6 ± 1.1, 1–6	**0.033**
TICI final, n (%)			0.552
0–2a	3 (2.7)	0 (0)	
2b, 3	110 (97.3)	57 (100)	
TICI final ≥2c, n (%)	70 (61.9)	47 (82.5)	**0.008**
mRS at 90 days, n (%)			1
mRS ≤ 2	41 (36.3)	20 (35.1)	
mRS >2	72 (63.7)	37 (64.9)	
Mortality at 90, days, n (%)	35 (31)	13 (22.8)	0.285

* *Fisher exact test (categorical variables); Mann–Whitney U-test (metric variables). Significant p-value marked in bold*.

§ *Control CT 24 h after treatment was not undergone in two patients of group 4 × 20*.

*ASPECTS post, Alberta Stroke Program Early Computed Tomography Score 24 h after stroke; IQR, interquartile range; mRS, modified Rankin scale; mTE/aTE, aspiration and mechanic thrombectomy; TICI, modified thrombolysis in cerebral infarction; OR 95% CI, odds ratio und 95% confidence interval; PH, parenchymal hemorrhage; SAH, subarachnoid hemorrhage; SD, standard deviation; SICH, symptomatic intracranial hemorrhage*.

**Table 3:** Preset 5 × 40 vs. pRESET 4 × 20 in M1-MCA occlusions: multivariate analysis.

	**Full model**		Stepwise selection^*^	
**First-pass reperfusion (TICI ≥2c)**	**OR (95% CI)**	*p*-value^*^	**OR (95% CI)**	*p*-value^*^
pRESET 5 × 40 vs. 4 × 20	2.20 (1.08–4.48)	0.030	2.18 (1.07–4.44)	0.032
Age (years)	1.03 (1–1.06)	0.052	1.03 (1–1.06)	0.035
ASPECTS basal	1.18 (0.93–1.49)	0.173	—	—
Collaterals (angiography)	2.35 (1.15–4.76)	0.018	2.73 (1.37–5.43)	0.004
Time to recanalization	0.92 (0.84–1)	0.051	0.91 (0.83–0.99)	0.033
**First-pass reperfusion (TICI ≥2b)**	**OR (95% CI)**	***p***-value^*^	**OR (95% CI)**	***p***-value^*^
pRESET 5 × 40 vs. 4 × 20	2.11 (1.04–4.28)	0.037	2.11 (1.04–4.28)	0.037
Collaterals (angiography)	1.96 (1.01–3.77)	0.045	1.96 (1.01–3.77)	0.045
Time to recanalization	0.91 (0.84–0.98)	0.014	0.91 (0.84–0.98)	0.014
**Complete reperfusion, (TICI ≥2c)**	**OR (95% CI)**	***p***-value^*^	**OR (95% CI)**	***p***-value^*^
pRESET 5 × 40 vs. 4 × 20	2.47 (1.01–6.04)	0.047	2.38 (1.01–5.60)	0.047
Age (years)	2.39 (1.16–4.93)	0.018	—	—
No. of passes	0.64 (0.49–0.85)	0.002	0.62 (0.48–0.82)	0.001
Time to recanalization	0.99 (0.88–1.11)	0.862	—	—
Collaterals (angiography)	2.39 (1.16–4.93)	0.018	2.15 (1.06–4.37)	0.035

* *Statistics: logistic regression with forward selection (p-value threshold for adding a variable: 0.05). n = 169 (one patient had no valuable collaterals in angiography)*.

*ASPECTS, Alberta Stroke Program Early Computed Tomography Score; NIHSS, National Institute of Health Stroke Scale; OR, odds ratio; CI, confidence interval; mTICI, modified Treatment In Cerebral Infarction score*.

Variables associated with FP in bivariate analysis were thrombectomy with a 5 × 40 stent retriever, better collaterals in the angiography, absence of SAH, type of DAC used, older age, higher ASPECTS on baseline imaging and at 24 h, lower duration of treatment, and time to recanalization. mFP was associated with better collaterals in angiography, absence of SAH, type of DAC, higher ASPECTS after 24 h, lower duration of treatment, and lower time to recanalization. FP was also associated in bivariate analysis with good clinical outcome, OR = 2.28 (1.18–4.38), *p* = 0.016, whereas mFP associated more frequently with a good clinical outcome, OR = 1.94 (1–3.75), without reaching statistical significance.

Variables associated with a complete reperfusion (TICI ≥2c) in bivariate analysis were thrombectomy with a 5 × 40 stent retriever, better collaterals in the angiography, older age, higher ASPECTS at 24 h, lower number of passes with stent retriever, lower time from onset to groin puncture, lower time to recanalization, and lower duration of treatment. Complete reperfusion was not associated with good clinical outcome at 90 days in the whole population [in TICI ≥2c 39.3% vs. not TICI ≥2c 28.3% good clinical outcome, OR = 1.64 (95% CI = 0.81–3.34), *p* = 0.227]. But in patients with a prestroke mRS of 0, achieving a TICI ≥2c is associated with 71.4% of good clinical outcome at 90 days vs. achieving TICI 1–2b, which is associated with 38.1% of good clinical outcome at 90 days [OR = 4.06 (95% CI = 1.19–13.82), *p* = 0.024].

**Figure 2:**
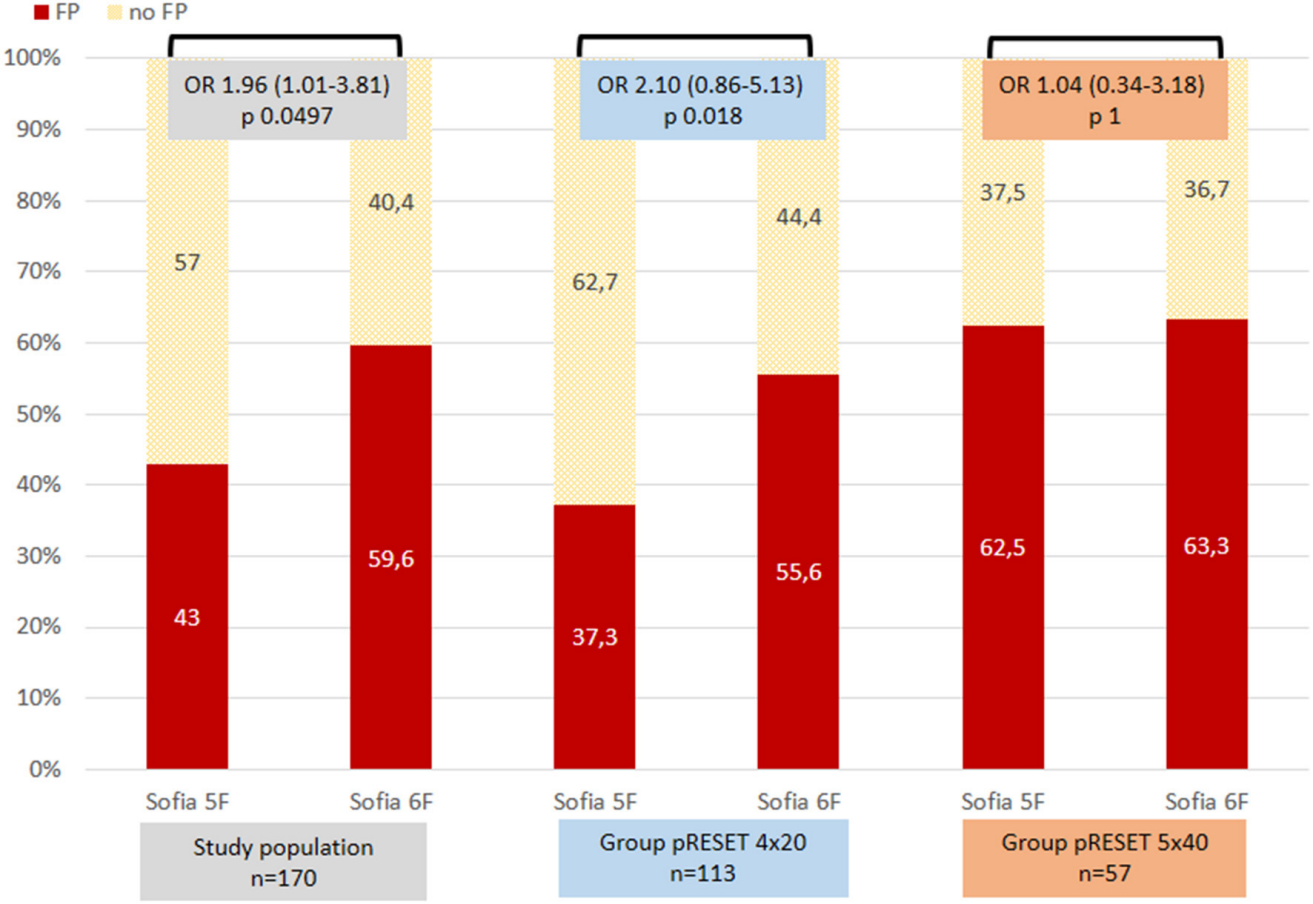
First-pass reperfusion (FP) regarding use of distal access catheter in the whole population and in groups 4 × 20 and 5 × 40. Statistics, Fisher exact test; odds ratio (OR) and 95% confidence interval (CI).

Regarding use of first DAC (Figure 2) and taking into account only Sofia, as the use of the other DACs is anecdotic, in the whole study population, we found higher FP with Sofia 6F (59.6%) vs. Sofia 5F (43%), *p* = 0.049. In the pRESET 4 × 20 group, using Sofia 6F as first DAC compared to Sofia 5F associated higher FP (55.6 vs. 37.3%, OR = 2.10, *p* = 0.018). In the pRESET 5 × 40 group, there was no difference regarding FP between both sizes of DAC. No differences were found between the type of first DAC used and rate of complete reperfusion (TICI ≥2c); although in the whole study population there was a higher frequency of complete reperfusion with Sofia 6F as first DAC used (78.9%) vs. Sofia 5F (64.5%), *p* = 0.074; 4 × 20 and 5 × 40, groups follow the tendency but without statistical significance.

The number of passes to achieve reperfusion was lower in the 5 × 40 group than in the 4 × 20 group (mean ± SD = 2 ± 1.4 vs. 1.6 ± 1.1, *p* = 0.033). In 39 patients of the 5 × 40 group, a single pass was sufficient. More than one pass was needed in 18 patients (31.5%) in the 5 × 40 group and in 55 patients (48.6%) in the 4 × 20 group. In bivariate analysis, a lower number of passes was associated with the use of the 5 × 40 device, successful and complete reperfusion, age, and ASPECTS, whereas a higher number of passes was associated with atrial fibrillation and the presence of SAH in control CT. After adjusting for relevant variables, the number of passes was not associated with thrombectomy with the 5 × 40 retriever [incidence rate ratio = 0.84 (95% CI = 0.69–1.03), *p* = 0.096].

Regarding clinical outcome and safety variables, we did not find differences between the 5 × 40 and 4 × 20 groups in mRS at 90 days, mortality, ASPECTS 24 h after treatment, PH, SICH, or SAH (Table 2). Incidence of vasospasm after thrombectomy was similar in both groups (27.4% in group 4 × 20 and 22.8% in group 5× 40) (Table 2). Only one patient in the 4× 20 group had a perforation of the MCA after the third pass of thrombectomy, and no embolization in a new territory or dissection in any group was reported.

A good clinical outcome at 90 days was achieved in 35.1% of patients in the 5 × 40 group and 36.3% in the 4 × 20 group (*p* non-significant). Variables associated with good clinical outcome were absence of atrial fibrillation, direct presentation in our hospital, better collaterals in angiography, absence of SAH, FP, younger age, higher ASPECTS prestroke and poststroke, lower NIHSS at baseline, lower number of passes, and lower time from imaging to groin puncture. FP was not independently associated with good outcome at 90 days in the whole study population in multivariante analysis. Taking into account only patients with an mRS prestroke 0–2, achieving an FP-associated good clinical outcome at 90 days occurred in 53.1% of patients, compared with not achieving FP, which resulted in good clinical outcome in only 31% of patients [OR (95% CI) = 2.27 (1.11–4.65), *p* = 0.024].

## Discussion

We found a significantly higher FP (TICI ≥2c) for M1 occlusions treated with thrombectomy under aspiration with longer stent retriever compared to shorter stent retriever, 61.4 vs. 40.7%, respectively. FP for all patients in both groups was 69.2%.

Although we assume that frequencies are not directly comparable because of the nature of each study and different patient characteristics, inclusion criteria, and techniques applied, our reported frequencies of FP are higher than in the published literature. FP frequency (TICI ≥2c) described for stent retriever in M1 occlusions was 39.7% (Trevo, almost in 90% of cases) (9). In the data of the NASA registry, for M1 occlusions, FP was 29% (Solitaire) (6), with younger and selected patients, in 2012 and 2013, and in the TRACK registry, FP was 22.3% for M1 occlusions (Trevo 4 × 20) (29). In the ASTER trial, FP in M1 occlusions was 31.2% with stent retriever or aspiration, within 6 h of onset (10). In the STRATIS registry, FP was 40% (Solitaire and Mindframe) (30). Recently, for LVO (including ICA, M1, M2, and posterior circulation), 53% was reported, and for M1, 51% in the ARISE II study [clinical trial with EmboTrap device (Cerenovus, Irvine, CA, USA) (8)]. Including ICA, MCA, and other LVO localizations, although with 85.6% of M1 occlusions, 22.8% of FP was reported in anterior circulation strokes within 6 h of stroke onset treated with only stent retriever, or aspiration or a combination of both (31). Some of these studies (9, 10) include tandem occlusions or intracranial stenosis, or in other studies, it is unknown, and these could be one factor influencing the difference of frequencies as these lesions preclude a FP because of the need for other maneuvers. If we had counted the patients with dissection or intracranial stenosis, FP frequency would still be high at 39% in the 4 × 20 group and at 50.7% in the 5 × 40 group.

Independent predictors of FP in the literature were use of BGC, better collateral grade (30, 32), site of occlusion with ICA-terminus occlusion as worse predictor and M1 occlusion as better predictor of FP (6, 30, 31), older age, lower systolic blood pressure, a higher DWI-ASPECTS at admission, local anesthesia, and combined first-line device strategy (31). We also found pial collaterals in the angiography, as an independent predictor of FP. Collaterals in acute stroke with LVO correlate with outcome; good collaterals are associated more likely with smaller core infarction, whereas poor collaterals associate larger core infarctions and more rapid infarct growth. Good collaterals also correlate with improved outcome after endovascular treatment and a favorable response to it, with higher recanalization rate and with less core infarct growth (33, 34). Angiographic collaterals were associated in our study with FP, mFP, good clinical outcome at 90 days, and complete reperfusion. Interobserver and intraobserver agreement of collateral circulation grading using the ASITN/SIR score was poor; a simplified dichotomized evaluation was considered more reproducible (23), why we decided to follow this suggestion. A possible explanation to a higher FP frequency with better leptomeningeal collaterals in the angiography could be that they provide a better definition of length of the thrombus, which allows a better position of the stent retriever related to the thrombus; also, good collaterals could avoid thrombus progression as they exert a retrograde pressure over the thrombus.

FP has been reported to be an independent factor for favorable clinical outcome (6, 7), with rates of 90-day better clinical outcome of 61.3% by FP TICI ≥2c (6) and rates of 67% by FP TICI 3 (7), and is associated with lower mortality rate, reduced hemorrhagic transformation, and procedural complications (10). Our study did not find an association between FP and clinical outcome, probably because of clinical characteristics of our population, with a large proportion of patients (21%) older than 85 years, almost 22% of patients with mRS prestroke >2, 10.6% with ASPECTS on baseline imaging ≤ 6 and long times from stroke onset to groin puncture and to recanalization. We observed that including patients with baseline mRS prestroke >2 influenced this result, as patients in our study with mRS prestroke 0–2 and FP had 53.1% of good clinical outcome at 90 days (good clinical outcome by FP and mRS prestroke 0, 71.4%; by mRS prestroke 1, 57.9%; by mRS prestroke 2, 17.6%), similar to previous publications.

Despite the observed association between size of stent retriever and higher frequency of FP, the size of retriever was not associated in our study with a favorable outcome at 90 days, and in our opinion, the size of the study population could be one reason. Likewise, the STRATIS registry even with a prestroke mRS ≥1 did not find an association between stent retriever size and clinical outcome or mortality by comparable final revascularization between groups (14).

Both clot control and aspiration are necessary for a successful endovascular treatment of LVOs. Clot control with stent retriever depends on multiple factors such as device mechanical characteristics, device behavior during retrieval, and thrombus biomechanics and consistencies (11).

The pRESET 4 × 20 stent retriever has shown a high recanalization rate experimentally *in vivo* (35) and in daily clinical practice (18, 36, 37). *In vitro* experience with the 4 × 20 pRESET and 6 × 30 pRESET has shown a close apposition to the vessel wall during thrombus removal, and highly effective clot removal for both white and red thrombi, with the 6 × 30 demonstrating a 60 and 100% rate of removal for white and red thrombi, respectively, the highest in comparison to other stent retrievers (14). If the specific device characteristics of the pRESET stent retriever play a role in achieving a higher FP, it cannot be elucidated from this study. A clinical comparison between pRESET 4 × 20 and 5 × 40 has to date not been published.

Retriever size could, as mentioned, influence stent properties. Longer and larger stent retrievers provide the potential to catch the clot on multiple planes of attachment in smaller arteries (2–3 mm) like the MCA and keep the engagement at larger vessel diameters in ICA, during clot retrieval (15). Larger-diameter stent retrievers come with higher radial force and a better vessel wall apposition. This results in more stability during the retrieval process (11). Previously, a comparison between 4- and 6-mm diameter Solitaire stent retrievers did not find differences in endovascular treatment outcomes for occlusions of the ICA, MCA, or ACA (16), focusing on reperfusion TICI ≥2b, not ≥2c, and without evaluation of FP. In our opinion, the superiority of the 5 × 40 over 4 × 20 might be rather related to the stent retriever length than to the stent retriever diameter. Longer stent retrievers have previously demonstrated *in vitro* higher rates of complete clot removal (11, 12).

Large white clots with higher fibrin content are stiffer and more difficult to retrieve in comparison to red blood cell–rich clots. Tests *in vitro* with white clot thrombus showed that stent retrievers up to 40 mm did not expand and therefore do not capture this type of thrombus if longer than 6 mm (11); for white thrombus between 2- and 4-mm, stent retrievers could expand, and thrombus was retrieved. *In vitro* studies with 20-mm-long fibrin-rich cloth have shown in M1 occlusion a frequency of FP for Solitaire 6 × 40 of 95%, and for 6 × 30, 67% (if a BGC was used, FP was 100% for both sizes, and if a 0.088-inch sheath and DAC catheter were used, FPs were 83% and 33%, respectively, for 6 × 40 and 6 × 30) (15).

The observed higher frequency of FP with longer stent retrievers in clinical practice has already been described. Longer stent retrievers (4 × 30 to 4 × 40) vs. short (4 × 20) were an independent predictor of mFP (TICI ≥2b) (13) in occlusions of intracranial ICA, M1, and M2 treated with BGC; they also included tandem occlusions; mFP was 62% in long and 50% in short stent retrievers, TICI 2b final was 98% in long and 94% in short stent retrievers with comparable clinical outcomes and comparable SAH. Zaidat et al. (14) reported the highest rate of FP and mFP in ICA, M1, and M2 occlusions with longer stent retrievers (4 × 40) compared to larger (6 × 30) or shorter (4 × 20) without differences in final revascularization or functional outcome. For MCA occlusions, they found the highest rates of mFP and FP with 4 × 40, 71.5 and 49.5%, respectively, and 6 × 30 did not perform better than 4 × 20 (mFP and FP, respectively, 63.2% and 41.6 vs. 59.5% and 41.3%) (14).

On the contrary, comparing lengths of stents (short: 4 × 20 mm and long >20 mm: 4 × 40, 6 × 30, or 4 × 30), Styczen et al. (17) found comparable FP TICI 3 for both short and long stent retrievers in MCA (50.8 vs. 40.7%) occlusions with higher mFP in short vs. long stent retrievers (50.8 vs. 40.7%, *p* = 0.024). But long retrievers achieved a higher rate of successful reperfusion (TICI ≥2b) with higher rate of SAH.

Possible explanations for higher rates of FP with longer stent retrievers are summarized here. As proximal and distal parts of the stent retriever are non-working, longer stent retrievers offer a longer working length, which potentially offer a larger surface for device integration in the clot and uniform distribution of forces along with the clot during traction (13). Using longer stent retrievers allows some degree of imprecision of placement (38) by lack of operator experience and allows for engagement of the entire thrombus in cases of vessel tortuosity/elongation when tension causes a proximal dislocation of the stent retriever by deployment, or if patients without general anesthesia move and make deployment imprecise. Also, a longer stent retriever, with a distal segment beyond the clot in M2, could help to anchor the stent retriever if the operator prefers to remove the microcatheter before the aspiration through DAC during the thrombectomy (38). The part of the retriever placed distal to the thrombus could help to sweep along a clot that does not integrate in the struts. Also, when a push-and-fluff technique is used for better wall apposition resulting in device foreshortening on active deployment, a longer retriever offers a higher security to cover the whole clot (39).

Achieving a reperfusion in only one pass of thrombectomy is our goal, but it is not always possible; therefore achieving a successful or complete reperfusion in the lower number of passes is also determinant. Reperfusion after fewer passes results in better outcomes in comparison to after a higher number of passes (6, 40). Multiple passes of thrombectomy are associated with worse outcomes and higher complication rates (41, 42). Thrombectomy attempts are associated with a risk for distal embolization and vessel damage. In our study, the number of passes is associated independently significant with higher frequency of SAH in control CT. In our study, thrombectomy with a longer stent retriever achieved reperfusion in a lower number of passes than the shorter stent retriever but after adjustment with other relevant variables lost significance. In both groups, the number of passes was low, and this could be one cause for not detecting a difference.

Theoretically, a longer retriever could have more contact to the vessel wall and cause more friction. Safety assessment of longer stents *in vivo* in porcine models has been done for 4-mm-diameter Solitaire devices, 4 × 40 and 4 × 20 (43), and in 6-mm devices, 6 × 40 and 6 × 30 (15), without difference in vascular safety parameters at 90 days. We found similar frequency of vasospasm and SAH after thrombectomy with both stent retriever sizes. No perforation was seen with pRESET 5 × 40. Our observations show that the longer stent retriever is safe to use for M1 occlusions.

Use of BGC was reported to be a predictor of FP (6, 30). The use of a BGC did not affect angiographic outcomes in other reported studies (29, 31). We achieved good FP results without the use of a BGC. We hypothesized that emboli appear when the microcatheter, stent retriever, and DAC are removed at the same time from M2-MCA to M1-MCA and to ICA as vessel diameter changes. We do not use a BGC for intracranial occlusions, with good angiographic and clinical results. As we almost always try to advance the DAC up to the M1 segment, we remove the stent retriever inside the DAC and leave the DAC under aspiration; then, under aspiration, we also remove the DAC minimizing risk of emboli in new territory as we avoid the loss of engagement of the clot during retrieval (44). Sometimes, we remove the stent retriever and the DAC at the same time under aspiration. Thrombus is retrieved within the stent retriever or within the DAC, after removal, or in both devices. Positioning of a BGC high enough in the ICA is difficult in patients with marked vessel elongation and increases risk of complication specially with a 9F BGD (allowing for the use of a 6F DAC). If the position of the BGC is not high enough, the DAC could be too short to be advanced up to intracranial ICA bifurcation. We agree that the use of BGC could be better in reducing distal emboli, but in our experience, it is not cost-effective.

As we try to advance the DAC over the stent, both stent retriever and aspiration have a role in reperfusion rate in our study. The use of a DAC was not independently associated with FP, although we achieved higher FP with the Sofia 6F in the 4 × 20 group. In the 5 × 40 stent retriever group, there was no difference in using both sizes of DAC (Figure 2). In our experience, we think a larger inner diameter DAC brings more lumen for aspiration but also could be associated with difficulties in navigating the DAC distally to the M1 segment. We accept that our FP rate is a result of a combined technique of stent retriever and aspiration, but as treatment in both groups was performed with exactly the same technique, we can assume that the size of stent retriever matters.

The retrospective nature of our study is a major limitation, and selection bias could have occurred. We have designed a prospective, randomized study comparing both size of retriever for M1 occlusions, and we look forward to verify our results. The single-center design could also be a limitation but may demonstrate the benefit of a standardized technique. The strengths of our study include a real life cohort, standardized technique, focus on M1 occlusions, and comparable patients in both groups; to reduce selection bias, all patients treated with 5 × 40 were included and compared to all patients treated with 4 × 20 at 1 year before.

## Conclusion

The size of the stent retriever matters in acute M1 occlusions treated with aspiration-assisted thrombectomy. A longer stent retriever achieves in one pass a higher first-pass complete reperfusion (TICI ≥2c) and first-pass successful reperfusion (TICI ≥2b) and higher complete reperfusion compared to a shorter stent retriever.

## Data Availability Statement

The raw data supporting the conclusions of this article will be made available by the authors, without undue reservation.

## Ethics Statement

The studies involving human participants were reviewed and approved by Ethik-Kommission der Landesäztekammer Baden-Württemberg. Written informed consent for participation was not required for this study in accordance with the national legislation and the institutional requirements.

## Author Contributions

CS: study conception, design of work, acquisition of data, interpretation of data, and drafting of manuscript. MA and VH: acquisition of data and critical revision of manuscript. HB: critical revision of manuscript. HH: study conception, design of work, and critical revision of manuscript. All authors contributed to the article and approved the submitted version.

## Conflict of Interest

CS, MA, and VH have consultancy agreements with phenox GmbH. HH is co-founder and shareholder of phenox GmbH. The remaining author declares that the research was conducted in the absence of any commercial or financial relationships that could be construed as a potential conflict of interest.

## References

[1] Powers WJ Rabinstein AA Ackerson T Adeoye OM Bambakidis NC Becker K. Guidelines for the early management of patients with acute ischemic stroke: 2019 update to the 2018 guidelines for the early management of acute ischemic stroke: a guideline for healthcare professionals from the American heart association/American stroke association. Stroke. (2019) 50:e344–418. 10.1161/STR.0000000000000211 31662037

[2] Goyal N Tsivgoulis G Frei D Turk A Baxter B Froehler MT. Comparative safety and efficacy of modified TICI 2b and TICI 3 reperfusion in acute ischemic strokes treated with mechanical thrombectomy. Neurosurgery. (2018) 83:593. 10.1093/neuros/nyy320 30010960

[3] Chamorro Á Blasco J López A Amaro S Román LS Llull L. Complete reperfusion is required for maximal benefits of mechanical thrombectomy in stroke patients. Sci Rep. (2017) 7:11636. 10.1038/s41598-017-11946-y 28912596 PMC5599658

[4] Liebeskind DS Bracard S Guillemin F Jahan R Jovin TG Majoie CB. eTICI reperfusion: defining success in endovascular stroke therapy. J Neurointerv Surg. (2019) 11:433–8. 10.1136/neurintsurg-2018-014127 30194109

[5] Dargazanli C Fahed R Blanc R Gory B Labreuche J Duhamel A. Modified thrombolysis in cerebral infarction 2c/thrombolysis in cerebral infarction 3 reperfusion should be the aim of mechanical thrombectomy: insights from the ASTER Trial (contact aspiration versus stent retriever for successful revascularization). Stroke. (2018) 49:1189–96. 10.1161/STROKEAHA.118.020700 29626134

[6] Zaidat OO Castonguay AC Linfante I Gupta R Martin CO Holloway WE. First pass effect: a new measure for stroke thrombectomy devices. Stroke. (2018) 49:660–6. 10.1161/STROKEAHA.117.020315 29459390

[7] Nikoubashman O Dekeyzer S Riabikin A Keulers A Reich A Mpotsaris A. True first-pass effect. Stroke. (2019) 50:2140–6. 10.1161/STROKEAHA.119.025148 31216965

[8] Zaidat OO Ribo M Mattle HP Saver JL Bozorgchami H Yoo AJ. Health economic impact of first-pass success among patients with acute ischemic stroke treated with mechanical thrombectomy: a United States and European perspective. J Neurointerv Surg. (2020) 21:016930. 10.1136/neurintsurg-2020-016930 33443119 PMC8606461

[9] Brehm A Maus V Tsogkas I Colla R Hesse AC Gera RG. Stent-retriever assisted vacuum-locked extraction (SAVE) versus a direct aspiration first pass technique (ADAPT) for acute stroke: data from the real-world. BMC Neurol. (2019) 19:65. 10.1186/s12883-019-1291-9 30987600 PMC6466709

[10] Ducroux C Piotin M Gory B Labreuche J Blanc R Ben Maacha M. First pass effect with contact aspiration and stent retrievers in the Aspiration versus Stent Retriever (ASTER) trial. J Neurointerv Surg. (2020) 12:386–91. 10.1136/neurintsurg-2019-015215 31471527 PMC7146919

[11] Machi P Jourdan F Ambard D Reynaud C Lobotesis K Sanchez M. Experimental evaluation of stent retrievers' mechanical properties and effectiveness. J Neurointerv Surg. (2017) 9:257–63. 10.1136/neurintsurg-2015-012213 27016318 PMC5339553

[12] Wenger K Nagl F Wagner M Berkefeld J. Improvement of stent retriever design and efficacy of mechanical thrombectomy in a flow model. Cardiovasc Intervent Radiol. (2013) 36:192–7. 10.1007/s00270-012-0420-2 22699778

[13] Haussen DC Al-Bayati AR Grossberg JA Bouslama M Barreira C Bianchi N. Longer stent retrievers enhance thrombectomy performance in acute stroke. J Neurointerv Surg. (2019) 11:6–8. 10.1136/neurintsurg-2018-013918 29858398

[14] Zaidat OO Haussen DC Hassan AE Jadhav AP Mehta BP Mokin M. Impact of stent retriever size on clinical and angiographic outcomes in the STRATIS stroke thrombectomy registry. Stroke. (2019) 50:441–7. 10.1161/STROKEAHA.118.022987 30626287

[15] Girdhar G Epstein E Nguyen K Gregg C Kumar T Wainwright J. Longer 6-mm diameter stent retrievers are effective for achieving higher first pass success with fibrin-rich clots. Interv Neurol. (2020) 8:187–95. 10.1159/000499974 32508901 PMC7253866

[16] Yang D Hao Y Zi W Wang H Zheng D Li H. Effect of retrievable stent size on endovascular treatment of acute ischemic stroke: a multicenter study. AJNR Am J Neuroradiol. (2017) 38:1586–93. 10.3174/ajnr.A5232 28596196 PMC7960417

[17] Styczen H Huseynov E Abdullayev N Maus V Borggrefe J Goertz L. Adjustment of stent retriever length to clot extent affects first-pass reperfusion in endovascular treatment of acute ischemic stroke. Cerebrovasc Dis. (2020) 49:277–84. 10.1159/000508028 32544906

[18] Kurre W Aguilar-Pérez M Schmid E Sperber W Bäzner H Henkes H. Clinical experience with the pREset stent retriever for the treatment of acute ischemic stroke–a review of 271 consecutive cases. Neuroradiology. (2014) 56:397–403. 10.1007/s00234-014-1346-y 24619135

[19] Adams HP Jr Bendixen BH Kappelle LJ Biller J Love BB Gordon DL. Classification of subtype of acute ischemic stroke. Definitions for use in a multicenter clinical trial. TOAST. Trial of Org 10172 in Acute Stroke Treatment. Stroke. (1993) 24:35–41. 10.1161/01.str.24.1.35 7678184

[20] Barber PA Demchuk AM Zhang J Buchan AM. Validity and reliability of a quantitative computed tomography score in predicting outcome of hyperacute stroke before thrombolytic therapy. ASPECTS Study Group. Alberta Stroke Programme Early CT Score. Lancet. (2000) 355:1670–4. 10.1016/s0140-6736(00)02237-6 10905241

[21] Kleine JF Beller E Zimmer C Kaesmacher J. Lenticulostriate infarctions after successful mechanical thrombectomy in middle cerebral artery occlusion. J Neurointerv Surg. (2017) 9:234–9. 10.1136/neurintsurg-2015-012243 26940316

[22] Lima FO Furie KL Silva GS Lev MH Camargo EC Singhal AB. The pattern of leptomeningeal collaterals on CT angiography is a strong predictor of long-term functional outcome in stroke patients with large vessel intracranial occlusion. Stroke. (2010) 41:2316–22. 10.1161/STROKEAHA.110.592303 20829514 PMC4939434

[23] Ben Hassen W Malley C Boulouis G Clarençon F Bartolini B Bourcier. Inter- and intraobserver reliability for angiographic leptomeningeal collateral flow assessment by the American society of interventional and therapeutic neuroradiology/society of interventional radiology (ASITN/SIR) scale. J Neurointerv Surg. (2019) 11:338–41. 10.1136/neurintsurg-2018-014185 30131382

[24] Zaidat OO Lazzaro MA Liebeskind DS Janjua N Wechsler L Nogueira RG. Revascularization grading in endovascular acute ischemic stroke therapy. Neurology. (2012) 79:S110–6. 10.1212/WNL.0b013e3182695916 23008384 PMC4109231

[25] Goyal M Fargen KM Turk AS Mocco J Liebeskind DS Frei D. 2C or not 2C: defining an improved revascularization grading scale and the need for standardization of angiography outcomes in stroke trials. J Neurointerv Surg. (2014) 6:83–6. 10.1136/neurintsurg-2013-010665 23390038 PMC4156591

[26] Kaesmacher J Dobrocky T Heldner MR Bellwald S Mosimann PJ Mordasini P. Systematic review and meta-analysis on outcome differences among patients with TICI2b versus TICI3 reperfusions: success revisited. J Neurol Neurosurg Psychiatry. (2018) 89:910–7. 10.1136/jnnp-2017-317602 29519899 PMC6109240

[27] Saver JL. Novel end point analytic techniques and interpreting shifts across the entire range of outcome scales in acute stroke trials. Stroke. (2007) 38:3055–62. 10.1161/STROKEAHA.107.488536 17916765

[28] Hacke W Kaste M Fieschi C von Kummer R Davalos A Meier D. Randomised double-blind placebo-controlled trial of thrombolytic therapy with intravenous alteplase in acute ischaemic stroke (ECASS II). Lancet. (1998) 352:1245–51. 10.1016/s0140-6736(98)08020-9 9788453

[29] Mokin M Primiani CT Castonguay AC Nogueira RG Haussen DC English JD. First pass effect in patients treated with the trevo stent-retriever: A TRACK registry study analysis. Front Neurol. (2020) 11:83. 10.3389/fneur.2020.00083 32132966 PMC7040359

[30] Jadhav A Zaidat O Noqueira R Mueller-Kronast N Jovin T Liebeskind D. Predictors of first pass effect with neurothrombectomy for acute ischemic stroke. JNIS. (2019) 11(Suppl. 1):A1–134. 10.1136/neurintsurg-2019-SNIS.1 34784741

[31] Di Maria F Kyheng M Consoli A Desilles JP Gory B Richard S. Identifying the predictors of first-pass effect and its influence on clinical outcome in the setting of endovascular thrombectomy for acute ischemic stroke: Results from a multicentric prospective registry. Int J Stroke. (2021) 16:20–8. 10.1177/1747493020923051 32380902

[32] Srivatsa S Duan Y Sheppard JP Pahwa S Pace J Zhou X. Cerebral vessel anatomy as a predictor of first-pass effect in mechanical thrombectomy for emergent large-vessel occlusion. J Neurosurg. (2020) 24:1–9. 10.3171/2019.11.JNS192673 31978878

[33] Heit JJ Zaharchuk G Wintermark M. Advanced neuroimaging of acute ischemic stroke: penumbra and collateral assessment. Neuroimaging Clin N Am. (2018) 28:585–97. 10.1016/j.nic.2018.06.004 30322595

[34] Bang OY Saver JL Kim SJ Kim GM Chung CS Ovbiagele B. Collateral flow predicts response to endovascular therapy for acute ischemic stroke. Stroke. (2011) 42:693–9. 10.1161/STROKEAHA.110.595256 21233472 PMC3051344

[35] Mordasini P Brekenfeld C Byrne JV Fischer U Arnold M Jung S. Experimental evaluation of immediate recanalization effect and recanalization efficacy of a new thrombus retriever for acute stroke treatment *in vivo*. AJNR Am J Neuroradiol. (2013) 34:153–8. 10.3174/ajnr.A3275 22837308 PMC7966317

[36] Prothmann S Schwaiger BJ Gersing AS Reith W Niederstadt T Felber A. Acute recanalization of thrombo-embolic ischemic stroke with pREset (ARTESp): the impact of occlusion time on clinical outcome of directly admitted and transferred patients. J Neurointerv Surg. (2017) 9:817–22. 10.1136/neurintsurg-2016-012556 27530601 PMC5574392

[37] Schwaiger BJ Kober F Gersing AS Kleine JF Wunderlich S Zimmer C. The pREset stent retriever for endovascular treatment of stroke caused by MCA occlusion: safety and clinical outcome. Clin Neuroradiol. (2016) 26:47–55. 10.1007/s00062-014-0329-z 25112831 PMC4833806

[38] Ospel JM Volny O Jayaraman M McTaggart R Goyal M. Optimizing fast first pass complete reperfusion in acute ischemic stroke - the BADDASS approach (BAlloon guiDe with large bore Distal Access catheter with dual aspiration with Stent-retriever as Standard approach). Expert Rev Med Devices. (2019) 16:955–63. 10.1080/17434440.2019.1684263 31648562

[39] Haussen DC Rebello LC Nogueira RG. Optimizating clot retrieval in acute stroke: the push and fluff technique for closed-cell stentrievers. Stroke. (2015) 46:2838–42. 10.1161/STROKEAHA.115.010044 26374483

[40] Kharouba R Gavriliuc P Yaghmour NE Gomori JM Cohen JE Leker RR. Number of stentriever passes and outcome after thrombectomy in stroke. J Neuroradiol. (2019) 46:327–30. 10.1016/j.neurad.2019.03.014 30981826

[41] Baek JH Kim BM Heo JH Nam HS Kim YD Park H. Number of stent retriever passes associated with futile recanalization in acute stroke. Stroke. (2018) 49:2088–95. 10.1161/STROKEAHA.118.021320 30354993

[42] García-Tornel Á Requena M Rubiera M Muchada M Pagola J Rodriguez-Luna D. When to stop. Stroke. (2019) 50:1781–8. 10.1161/STROKEAHA.119.025088 31177974

[43] Wainwright JM Jahan R. Solitaire FR revascularization device 4 × 40: safety study and effectiveness in preclinical models. J Neurointerv Surg. (2016) 8:710–3. 10.1136/neurintsurg-2015-011856 26101268

[44] Kurre W Vorlaender K Aguilar-Pérez M Schmid E Bäzner H Henkes H. Frequency and relevance of anterior cerebral artery embolism caused by mechanical thrombectomy of middle cerebral artery occlusion. AJNR Am J Neuroradiol. (2013) 34:1606–11. 10.3174/ajnr.A3462 23471019 PMC8051447

